# Isolation and characterization of nanocrystalline cellulose from cocoa pod husk (CPH) biomass wastes

**DOI:** 10.1016/j.heliyon.2021.e06680

**Published:** 2021-04-11

**Authors:** Adebola Iyabode Akinjokun, Leslie Felicia Petrik, Aderemi Okunola Ogunfowokan, John Ajao, Tunde Victor Ojumu

**Affiliations:** a Department of Chemical Engineering, Cape Peninsula University of Technology, Cape Town, South Africa; b Department of Chemistry, Obafemi Awolowo University, Ile-Ife, Nigeria; c Department of Chemical Sciences, Joseph Ayo Babalola University, Ikeji-Arakeji, Nigeria

**Keywords:** Cocoa pod, Wastes, Cellulose, Lignin, Cellulose nanocrystals, Hydrolysis

## Abstract

In this paper, cellulose nanocrystals (CNCs) were isolated from the cellulose extracted from cocoa pod husk waste, followed by characterization using XRD, FT-IR, TGA, SEM and TEM to determine its crystallinity, structural properties, thermal characteristics, morphology and dimensions respectively. The result revealed that the cocoa pod husk amorphous segments containing hemicelluloses and lignin were extensively removed with increasing chemical treatments leading to increased purity, crystallinity index and thermal stability of the extracted materials. The diameter, length and crystallinity index of the CNC isolated from the CPH are 10–60 nm, 41–155 nm and 67.60% respectively.

## 1 Introduction

Cocoa (*Theobroma cacao*) is an important fruit crop cultivated for its seed “*cocoa beans*” which is in high demand for use in the manufacture of chocolate, cocoa powder and butter. It is mostly cultivated in the Americas, Asia and West Africa (Dahunsi et al., 2019; Wessel and Quist-Wessel, 2015). Ivory Coast, Ghana, Nigeria and Cameroun, the four West African producers accounted for about 70% of global production in 2018 (Dahunsi et al., 2019). Of these four West African countries, Ivory Coast and Ghana alone account for more than 50% of global cocoa output annually. Nigeria, the 4^th^ largest on the average account for about 367,000 tons annually (Lateef et al., 2008). Alongside the important cocoa beans, massive biomass in the form of cocoa pod husk (CPH) is generated as waste (Aikpokpodion and Adeogun, 2011; Dahunsi et al., 2019). It has been estimated that more than 8 million tons of CPH biomass are generated annually in Nigeria (Lateef et al., 2008). Despite the huge potential of converting these wastes into valuable materials suitable for commercialization, virtually all is left to decay or burned in the harvest field.

Cocoa pod husk biomass represents about 75% of the cocoa fruit weight; for every ton of cocoa beans produced about 10 tonnes of cocoa pod biomass is generated as waste (Bello et al., 2011; Daud et al., 2013; Vriesmann et al., 2011). However, this massive feedstock supply which is currently underutilized could provide a continuous supply of raw material for a potential CPH biomass-based industry. The new potential industry could convert this biomass waste into value added materials such as fertilizer, composite materials, pulp among others (Daud et al., 2013). CPH biomass is a lignocellulosic material consisting of cellulose, hemicellulose, lignin, pectin, oils and waxes (Acebo-Guerrero et al., 2012; Igbinadolor and Onilude, 2013; Lu et al., 2018). Among the aforementioned components of the CPH biomass, cellulose accounts for the largest share (Lee et al., 2014; Singh et al., 2016) thus providing a feedstock from which sustainable value-added materials such as nanocrystalline cellulose (CNC) can be produced. Cellulose is a polymer of (C_6_H1_0_O_5_)_n_ linked by β-(1,4) glucosidic bonds and shows a regular network of intra and intermolecular hydrogen bonding which are organized into microfibrils (Collazo-Bigliardi et al., 2018; Lamaming et al., 2015; Mueller et al., 2014; Sheltami et al., 2012). Microfibrils are arranged such that it contained repeated and interwoven units of crystalline and amorphous segments. Chemical treatment of the cellulose fibers has been known to dissolve the amorphous domain yielding cellulose nanocrystals (CNCs) (Lamaming et al., 2015).

Cellulose nanocrystals are rod-like nanometric particles with at least one dimension less than or equal to 100 nm. CNCs have found wide potential applications as barrier films, in pharmaceuticals, slow drug release, paper, photonics and separation membranes (Henrique et al., 2013). In addition, CNCs have also found application as reinforcement agents in polymer composites (Klemm et al., 2005). Polymers reinforced with CNCs are known to display superior mechanical, thermal and barrier properties in comparison to those reinforced by conventional polymers (Henrique et al., 2013).

CNC derived from renewable feedstock have several advantages including low density, biocompatibility and biodegradability, non-toxicity and low cost (Dufresne, 2003; Habibi et al., 2010). Sources of cellulose used in the isolation of CNC is very important; it determines the dimensions and properties of CNC (Kargarzadeh et al., 2017) Isolation of CNC from cocoa pod husk biomass is therefore relevant (Neto et al., 2013) as it would significantly increase the sustainability of cocoa production. In addition, it would proffer solutions to the myriad of problems associated with CPH disposal (Chandra et al., 2016) and provide additional source of income to farmers.

Numerous studies on the extraction of CNC from lignocellulosic resources such as wood (Beck-Candanedo et al., 2005), pineapple leaves (Ravindran et al., 2019), oil palm biomass (Haafiz et al., 2014), mango seed (Henrique et al., 2013), banana pseudostem (Mueller et al., 2014), rice husk (Collazo-Bigliardi et al., 2018; Johar et al., 2012), sugarcane bagasse (Kumar et al., 2014), coffee husk (Collazo-Bigliardi et al., 2018), mengkuang leaves (Sheltami et al., 2012) and agave augustifolia (Rosli et al., 2013) among others have been reported in literature. The conventional and by far the most popular method reported for the isolation of CNC is the chemo-mechanical method comprising of a combination of acid hydrolysis followed by ultrasonication. According to Brito et al. (2012),the dimension (length and width) of CNC is determined by the acid hydrolysis condition and the source of cellulose used. The use of 60 wt.% (Rosli et al., 2013; Sheltami et al., 2012; Tonoli et al., 2012) and 64 wt.% (Fortunati et al., 2013; Haafiz et al., 2014; Lamaming et al., 2015) sulphuric acid (H_2_SO_4_) have been widely reported in the isolation of CNCs from different cellulose substrates. Few researchers have attempted the isolation of cellulose nanocrystals from cocoa pod husk waste. Jimat and Jami (2016), attempted the isolation of CNC from cocoa pod husk cellulose using only ultrasonication as an alternative method of isolating CNCs. This effort produced aggregates of microcrystalline cellulose (MCC) fibrils with 1405 nm–286 nm size distribution. Recently, a modified form of the conventional method in which mild sulphuric acid (1% v/v) was used for the hydrolysis of CPH has been reported (Jimat et al., 2020). In this attempt, CNCs with particle size distribution 200–400 nm were obtained. This paper presents the isolation of cellulose nanocrystals from cellulose extracted cellulose from cocoa pod husk waste using for the first time to the best of our knowledge the conventional chemo-mechanical method: acid hydrolysis (with 64 wt. % H_2_SO_4_) followed by ultrasonication. The effects of the chemical treatment on the cocoa pod husk and the resultant fibers obtained were characterized using Fourier transform infra-red (FTIR) spectroscopy, X-ray diffraction (XRD), Scanning electron microscopy (SEM), transmission electron microscopy (TEM) and thermogravimetric analysis (TGA).

## 2 Materials and methods

### 2.1 Materials

Dried cocoa pod husk biomass was collected from a cocoa plantation at Owena Ondo State, Nigeria. In this study, the following reagents were used: sodium hydroxide (Kimix, SA), ethanol, toluene, sodium chlorite (NaClO_2_ technical grade, 80%, Sigma Aldrich), glacial acetic acid sulphuric acid (Sigma-Aldrich 95%w/v), Cellulose dialysis membrane (Sigma Aldrich) and cellulose powder (B & M Scientific SA).

### 2.2 Extraction of cellulose

Cocoa pod husks (CPH) were washed several times with distilled water and dried in an oven at 80 °C for 24h before grinding into powder using a laboratory mill (Warring). The cocoa pod husk powder was then de-waxed in a boiling mixture of ethanol and toluene (1:2) for 6h. The de-waxed fibre was thereafter washed with ethanol and dried. A treatment procedure described in literature (Fortunati et al., 2013; Henrique et al., 2013; Neto et al., 2013) was subsequently followed for the extraction of the CPH cellulose. CPH fibre was firstly treated with 4% (w/v) NaOH solution at 80 °C for 2h, followed by bleaching with 1.7 wt. % NaOCl_2_ solution (whose pH has been adjusted to 4 by means of acetic acid) at 80 °C for 4h to obtain the cellulose fibre. The reaction mixture in each case was allowed to cool to room temperature, thereafter the residue was washed with distilled water. The cellulose fibre was dried in the oven at 60 °C for 24h. The ratio of the liquor to CPH was 5: 100 (g/mL).

### 2.3 Isolation of cellulose nanocrystals

CNCs were produced from the treated cocoa pod husk fibre by sulphuric acid hydrolysis protocol previously described elsewhere (Haafiz et al., 2014; Kallel et al., 2016). Hydrolysis was carried out with 64 wt. % H_2_SO_4_ at 45 °C for 60 min with vigorous stirring. At the end of the time stipulated for acid hydrolysis, the hydrolysed mixture was diluted tenfold with cold water to quench the reaction. The suspension obtained was centrifuged at 4500 rpm for 20 min to concentrate the cellulose nanocrystals and also to remove the excess acid. The resultant precipitate was re-dissolved in distilled water, recentrifuged, transferred into cellulose dialysis membrane and dialysed against deionized water until a neutral pH (pH 7) was obtained. The thick suspension obtained was sonicated at 40% output (while cooling in ice) for 10 min in a Misonix Ultra Liquid Processors (United Scientific). The ultrasonicated suspension was thereafter freeze dried to obtain the CNC powder.

### 2.4 Characterization

#### 2.4.1 X-ray diffraction (XRD)

X-ray diffraction (XRD) characterization of residues obtained at the end of each chemical treatment was carried out using a D8 Advance (Bruker AXS, Germany) XRD diffractometer fitted with a position sensitive Lynx Eye detector and equipped with a monochromatic Cu Kα radiation source (λ = 0.154nm) with the step scan mode set to 5^o^ to 50^o^ 2θ range. The equipment was operated at a potential of 40 kV and a current of 40 mA. The crystallinity index CrI, of samples were obtained according to the using the method described by Segal et al. (1959). Quantification of Crystallinity index is as shown in Eq. (1)

(1) $$\text{CrI}=\frac{{\text{I}}_{002}-{\text{I}}_{\text{amp}}}{{\text{I}}_{002}}\times 100$$

where *I*_002_ and is the intensity of the maximum diffraction peaks associated with the (002) crystalline lattice at 22 to 23 2θ degree and *I*_amp_ is the minimum diffraction peaks associated with the amorphous portion at 18 to 19 2θ degree. Data were also collected for commercial cellulose for comparison with those obtained for the CPH fibres.

#### 2.4.2 Fourier transform infrared (FT-IR) spectroscopy

Fourier transform infrared (FT-IR) spectra of the raw CPH, chemical treated CPH (CPC) and cellulose nanocrystals (CNCs), were recorded using an Attenuated Total Reflectance (ATR) PerkinElmer 400 FTIR/FT-NR spectrometer. This was to monitor changes in structural characteristics of the CPH samples before and after chemical treatments. Approximately 15 mg of each sample was placed on the diamond sample holder to facilitate the collection of infrared spectrum data. This was done by applying a gentle force to the sample by means of an adjustable knob attached to the sample holder. Infrared data collection was carried out by scanning samples from 4000 to 650 cm^−1^ at 4 cm^−1^ resolution and 50 scans for each sample. Baseline was corrected for background noise and smoothed before data collection.

#### 2.4.3 Thermogravimetric analysis (TGA)

The thermal stability of the raw cocoa pod fibre, chemically treated fibre and cellulose nanocrystals were characterized using a thermogravimetric analyser (Pyris instrument 4000, USA). Approximately 2 mg of sample was placed on the aluminium pan and heated from room temperature to 600 °C under a nitrogen environment at a heating rate of 10 ^o^/min and flow rate of 60 mL/min.

#### 2.4.4 Imaging analysis

##### 2.4.4.1 Scanning electron microscopy (SEM)

Surface morphology of samples was characterized using a Zeiss Gemini Auriga Scanning Electron Micro-analyser equipped with a CDU-lead detector at 25 kV. Small amount of each sample was placed on a carbon adhesive tape fixed onto an aluminium stub and sputter coated with gold. Samples were introduced into the analyzer and electron micrographs of each sample were captured at different magnifications.

##### 2.4.4.2 Transmission electron microscopy (TEM)

Transmission electron microscopy (TEM) images of CNCs were recorded on Tecnai TF20 HRTEM operating at 200 kV. A drop of diluted CNC suspension was placed on a copper grid and left to dry for 5 min. The grid was thereafter stained with 2% uranyl acetate solution for about 5 min and then dried before analysis.

## 3 Results and discussion

### 3.1 X-ray diffraction (XRD) measurement

**Figure 1:**
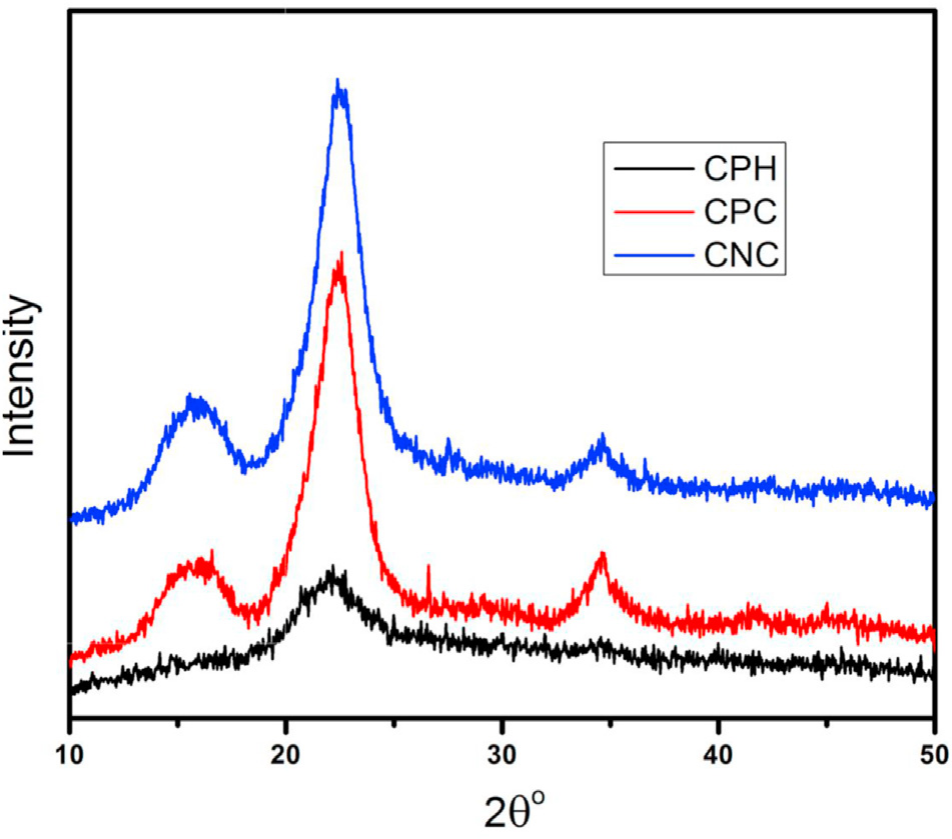
X-ray diffraction pattern for raw cocoa pod husk (CPH), cocoa pod cellulose (CPC) and cellulose nanocrystal (CNC).

Figure 1 shows the XRD diffractograms of raw cocoa pod husk powder (CPH), cellulose extracted from cocoa pod husk (CPC) and cellulose nanocrystals isolated from the cellulose (CNC). The main reflective peak in CPH, CPC and CNC is centred on 2θ > 22^o^ and corresponds to the (002) crystallographic plane. This is the only peak (2θ = 22.06^o^) observed in the reflective pattern of the raw CPH. This could be due to the presence of amorphous cementing materials covering the crystalline portion of the fibre. After alkaline treatment and bleaching of the CPH, to obtain CPC, two new crystalline peaks emerged in the diffractogram of CPC at 2θ = 15.82^o^ and 34.63^o^. CPC therefore show 3 peaks at 15.82^o^, 22.48^o^ and 34.63^o^ similar to what has been reported for the (110), (002) and (023) crystallographic planes of cellulose I polymorph thus indicating that CPC has typical cellulose I structure (Rosli et al., 2013). It is observed from Figure 1, that the peak at 2θ = 22.48^o^ in CPC diffractogram is narrower and have a higher intensity compared to that of raw CPH. This could be due to the dissolution of the amorphous hemicelluloses and lignin components of the CPH; subsequent recovery of the crystalline portion of the fibre resulted in the more intense and narrower peaks seen in CPC diffractogram. CNC isolated by acid hydrolysis, also show 3 peaks at 2 θ = 15.97^o^, 22.63^o^ and 34.63^o^. However, a slight change in position of peaks is seen in addition to increased intensity and narrowing of peaks. The amorphous background is characterized by low diffraction intensity around 2θ = 18^o^) (Segal et al., 1959). The crystallinity index, CrI, of raw cocoa pod husk powder, cellulose extracted from CPH and cellulose nanocrystals isolated from the CPH were found to be 33.93%, 63.31% and 67.60% respectively (Table 1). This clearly shows that the crystallinity of the isolated materials increases with progressive chemical treatments. This increase in crystallinity index is due to the removal of the amorphous constituents and the rearrangement of the crystalline regions into more ordered structure (Rosli et al., 2013). The progressive increase in CrI seen in this study is similar to the trend noticed during the isolation of CNC from mulberry bark (Li et al., 2009), agave angustifolia (Rosli et al., 2013), and oil palm trunk (Lamaming et al., 2015).

**Table 1:** Crystallinity index of cocoa pod husk fibres at different Stages of chemical treatment.

Sample	2θ (002)^o^	2θ (amorphous)^o^	Crystallinity index (CrI) (%)
CPH	22.06	18.05	33.93
CPC	22.48	18.17	63.31
CNC	22.63	18.19	67.60

The CrI of the CNC isolated in this study (Table 1) is similar to that reported for CNCs extracted from *Mengkuang* leaves (69.5%) (Table 2) (Sheltami et al., 2012). The CrI of the CPH cellulose nanocrystal is lower than the values obtained for CNCs isolated from *Agave angustifolia* (82%), waste paper (75.9%), Eucalyptus Kraft pulp (76%) and Mulberry bark (73.5%). However, it is higher than 55.9% reported for the CNC extracted from Corn cob by Liu et al. (2016).

**Table 2:** Dimensions and characteristics of cellulose nanocrystals from various sources.

Source	Length (nm)	Diameter (nm)	Yield (%)	Crystallinity (%)	Maximum degradation Temp. (^o^C)	Shape	References.
Cocoa pod husk	95 (average)	10–60 (26 average)		67.6	332	Rod-like	This study
*Mengkuang* Leaves	200 (average)	5–25	10.4	69.5	-	Needlelike	(Sheltami et al., 2012)
*Agave augustifolia*	310	8–15	-	82	361	Needlelike	(Rosli et al., 2013)
*Phormium tenax*	140-150 (average)	10–100	35	-	355	-	(Fortunati et al., 2013)
Eucalyptus kraft pulp		15 ± 6 (average)		76	525		(Tonoli et al., 2012)
Mulberry barks	400–500	20–40	-	73.4	335	-	(Li et al., 2009)
Corn cob		60–330	34.5	55.9	313	Rod-like	(Liu et al., 2016)
Waste paper	100–300	3–10	19	75.9	-	Needlelike	(Danial et al., 2015)

### 3.2 FTIR spectroscopy analysis

**Figure 2:**
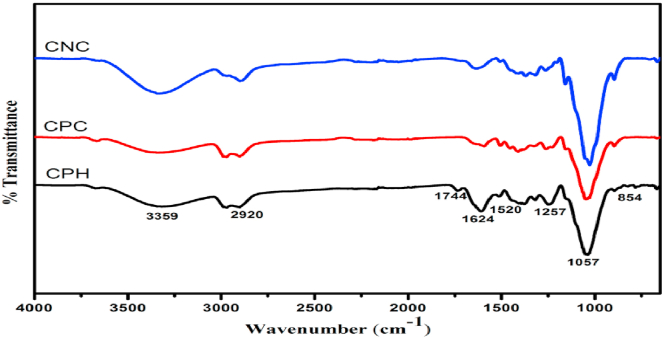
FTIR spectra of raw cocoa pod husk (CPH), alkali treated and bleached. cocoa pod husk (CPC) and acid hydrolysed cocoa pod husk (CNC).

Figure 2 shows the FTIR spectra of the raw cocoa pod husk (CPH), cellulose (CPC) and cellulose nanocrystals (CNC). In the spectrum due to the raw CPH, the band at 1744 cm^−1^ is ascribed to the stretching vibration of the acetyl and uronic ester groups in pectin, hemicelluloses or the ester linkage of carboxylic group of ferulic and p-coumaric acids of lignin and/or hemicelluloses (Costa et al., 2015; Rosli et al., 2013; Sheltami et al., 2012).This band is absent in the spectra of CPC and CNC obtained after chemical treatment and acid hydrolysis respectively. The disappearance of this band showed that all ester linkages of the hemicelluloses were cleaved by the alkali treatment. Ether linkages between lignin and hemicelluloses are however not affected by this treatment (Sheltami et al., 2012). The band at 1520 cm^−1^ and 1257 cm^−1^ in the spectra of CPH, CPC and CNC is attributed to the C= C in plane aromatic vibrations due to the presence of lignin and C–O–C ether stretching from ether linkage found in lignin. The presence of these bands in the spectra of CPC and CNC though with reduced intensity shows that the bleaching treatment did not effectively remove lignin from the CPH. The presence of lignin in the fibre after chemical treatment and acid hydrolysis could be responsible for the low crystallinity of CPC and CNC obtained from XRD calculations. The band at 1057 cm^−1^ seen in the spectra of CPH, CPC and CNC is due to C–O–C pyranose stretching skeletal vibration confirming the presence of cellulose in the three fibres. The intensity of this band can be seen to increase with progressive chemical treatments showing increase in crystallinity of the samples (Rosli et al., 2013). The band at 2920 cm^−1^ seen in the spectral of all samples is due to the C–H stretching vibration showing the presence of organic molecules in the samples while the bands at 854 cm^−1^ and 1624 cm^−1^ are due to C–H rocking vibration of carbohydrates and O–H bending vibration of water. The broad band with maxima at 3359 cm^−1^ is due to O–H vibration of adsorbed water molecule.

### 3.3 Thermogravimetric analysis

**Figure 3:**
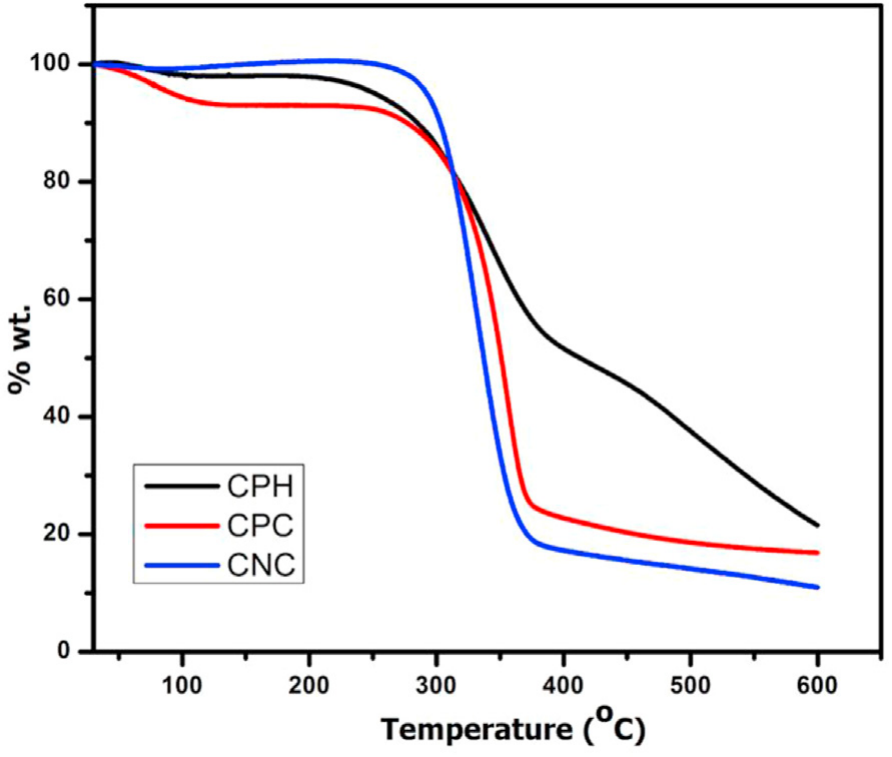
Thermogravimetric (TG) curves of raw cocoa pod husk (CPH), chemical treated CPH, (CPC) and acid hydrolysed CPC (CNC).

**Figure 4:**
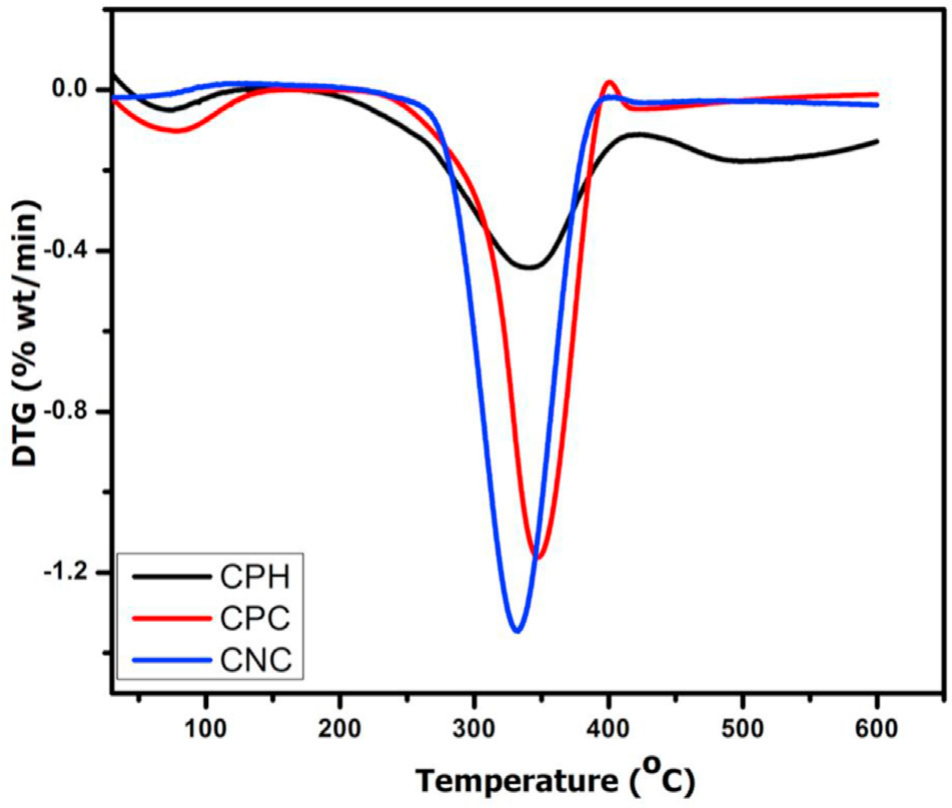
Differential thermogravimetric (DTG) curves of raw cocoa pod husk (CPH), chemical treated CPH, (CPC) and acid hydrolysed CPC (CNC).

Figures 3 and 4 shows the thermogravimetric (TG) analysis and the derived (DTG) plots of CPH, CPC and CNC. Both TG and DTG are presented as a function of temperature. The three samples present three main regions of weight loss. There is a slight decrease in % weight (clearly shown in the DTG) in all samples at 30 °C up to 120 °C with CPH showing the highest weight loss in this region and CNC the least. The decrease in % weight seen in this region has been linked to vapourization of adsorbed moisture on the surfaces of the samples as well as chemisorbed and H-bonded water molecules in the samples (Costa et al., 2015; Rosli et al., 2013; Sheltami et al., 2012). In addition, it could also be due to the volatilization of low molecular weight organics in the raw CPH and the residual hemicellulose in CPC (Morán et al., 2008). In the DTG curve for CNC (Figure 4), the peak associated with this weight loss is absent; this clearly shows that the low molecular volatile organic compounds associated with CPH and CPC were completely removed in the acid hydrolysed sample. From the TG curve in Figure 3, the thermal decomposition of the raw CPH began around 219 °C; on the DTG curve (Figure 4) the decomposition reached a maximum around 339 °C accounting for the pyrolysis of cellulose in the sample. In the case of the cellulose fibre, CPC obtained by alkali treatment and bleaching, thermal decomposition commenced at 256 °C; this is lower than those reported in literature for cellulose extracted from the bark of mulberry tree (Li et al., 2009), agave *augustifolia* (Rosli et al., 2013) and corn cob (Costa et al., 2015) but similar to that reported by Sheltami et al. (2012) for cellulose extracted from mengkuang leaves. The lower degradation onset temperature seen here for cellulose extracted from cocoa pod husk could be caused by the residual hemicellulose component left after the chemical treatment. The maximum peak in the DTG for the fibre obtained by chemical treatment, (CPC) is around 350 °C. CNC, obtained by acid hydrolysis of the cellulose exhibits higher start degradation temperature of 265 °C, with a sharp maximum peak seen in its DTG (Figure 4) at 332 °C. Decomposition at temperatures above 400 °C is associated with the pyrolysis of lignin; activities within this temperature is higher in the raw CPH followed by CPC and least in CNC due to the decreasing amount of lignin in the samples with progressive treatments.

The maximum thermal degradation temperature of the CNCs is similar to the values reported for CNCs isolated from *Phormium tenax*, mulberry fibres, and corn cob (Table 2).

### 3.4 Microscopy

**Figure 5:**
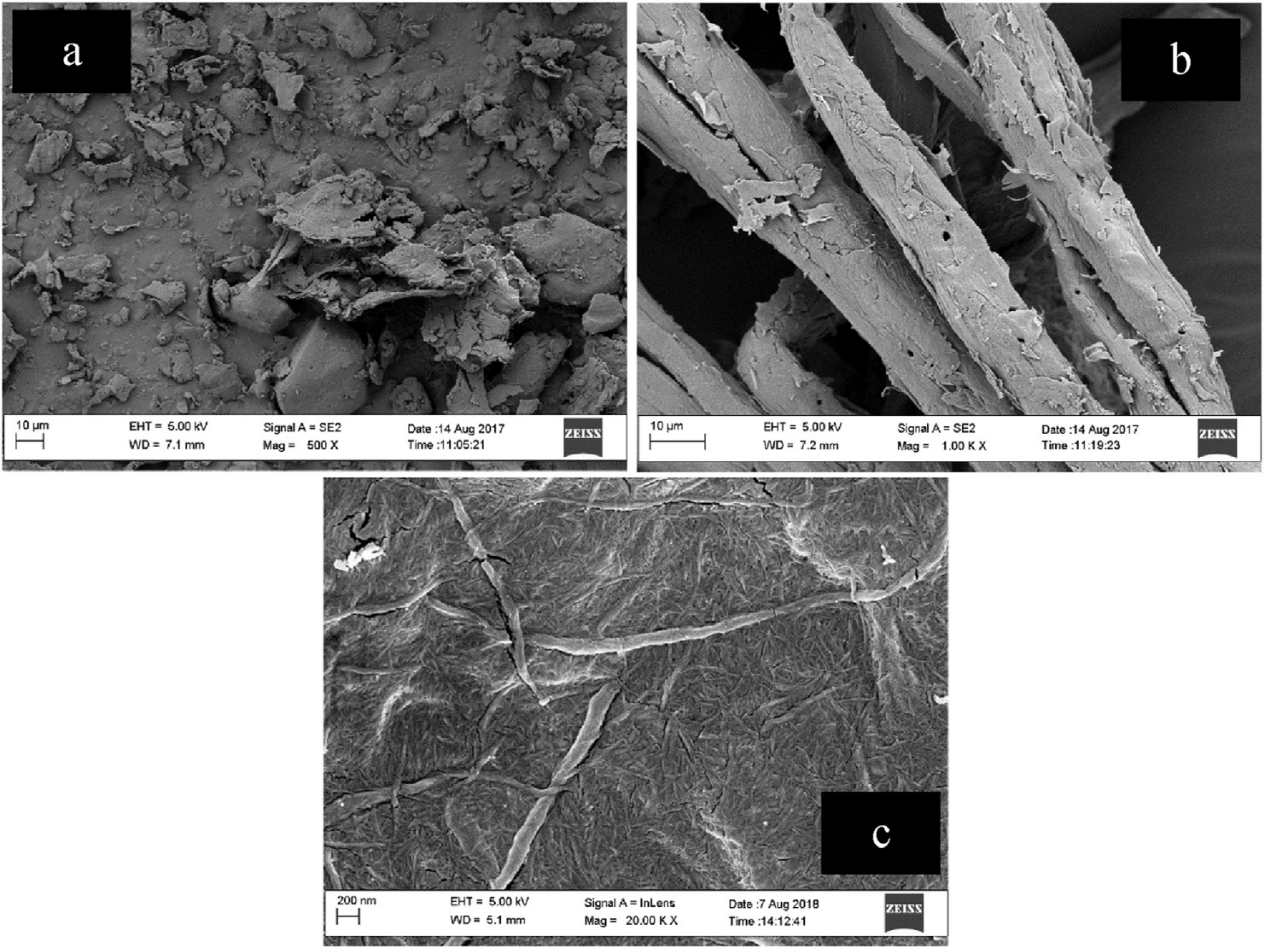
SEM images of (a) untreated cocoa pod husk (top left corner); (b) cellulose (top right corner) and (c) cellulose nanocrystals (bottom).

The morphology of the cocoa pod husk biomass at different stages of the chemical treatment was investigated by scanning electron microscope (SEM). Figure 5 shows the SEM micrographs at different stages of chemical treatment. With progressive chemical treatments, a noticeable change is seen in the morphology of the CPH. The individual lignocellulosic structure of the raw CPH(Figure 5a) is not visible as they are still layered or cemented by wax, hemicellulose, pectin, lignin among other impurities. Soxhlet extraction using a mixture of ethanol and toluene facilitated the removal hydrophobic waxes and pectin. Removal of these hydrophobic layers increased the accessibility of cellulose to chemical attack (Mazlita et al., 2016). The micrograph of the alkali treated and bleached cocoa pod husk (Figure 5b) appears smoother with distinct individual structure compared to the CPH fibre.During the alkali treatment, hemicelluloses are hydrolysed and become water soluble. Alkali treatment also facilitates defibrillation of the fibre structure (Figure 5b).

**Figure 6:**
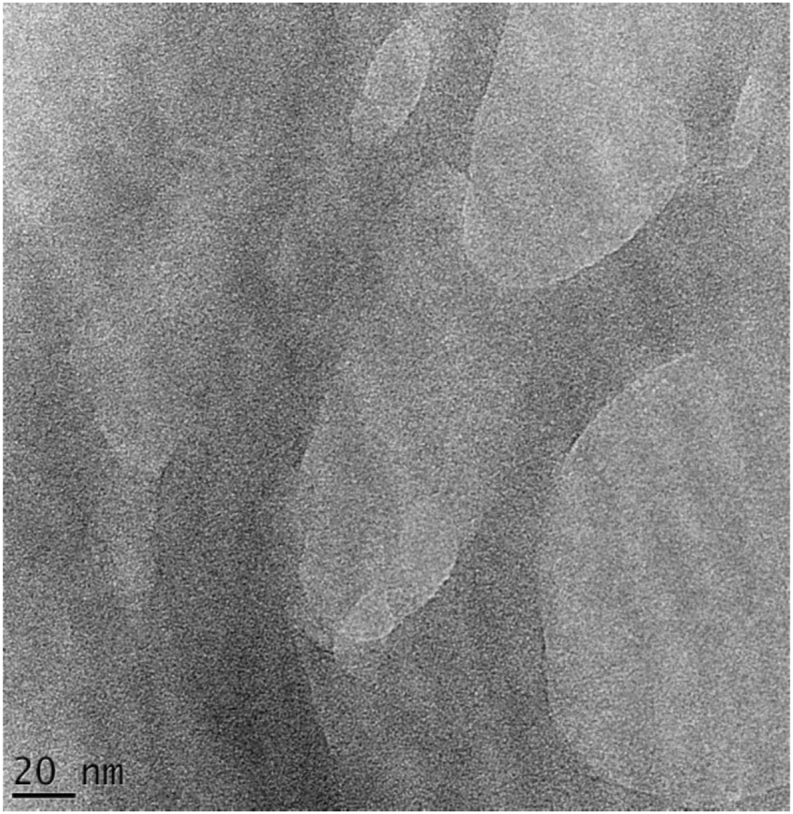
TEM image of CNC.

**Figure 7:**
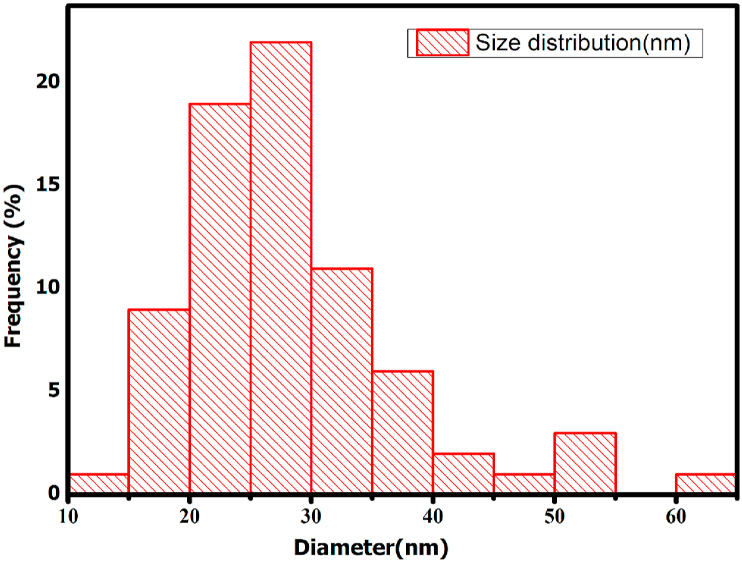
Size distribution of cellulose nanocrystals obtained from cocoa pod husk

The subsequent bleaching treatment resulted in further defibrillation and modification of the fibre surface. After bleaching, the acid hydrolysis removed the amorphous portion of the cellulose and broke the fibre bundles into individual cells (Figure 5c). Figure 5c and Figure 6 respectively shows the SEM and TEM micrograph of the acid hydrolysed cocoa pod husk cellulose. The dimensions and characteristics of CNCs isolated from other sources are summarized in Table 2. Transmission electron microscopy (TEM) image showed that the CNCs obtained from the acid hydrolysis of cocoa pod husk cellulose have a rod-like morphology (Figure 6) similar to that reported for corn cob. The diameter of the CNC was calculated and their distribution is as illustrated in Figure 7. The diameter of the CNCs ranged from 10 - 60 nm while the length ranged from 41-155 nm with an average of 26 nm and 95 nm respectively. From the diameter range of the CNC, it is obvious that the CNC isolated from CPH in this study are non-uniform and have widely dispersed sizes. This diameter is similar to the 10–100 nm diameter range reported for CNC obtained from *Phormium tenax* by Fortunati et al. (2013) and lower than the 60–330 nm reported for CNC isolated from corn cob by Li et al. (2009).However, the range is higher than the values reported for CNCs isolated from *Mengkuang* leaves (5–25 nm), *Agave angustifolia* (8–15 nm), mulberry fibres (20–40 nm) and waste paper (3–10 nm).

Furthermore, the average diameter of CNC produced in this study is higher than the 15 ± 6 nm reported for CNCs extracted from Eucalyptus Kraft pulp (Tonoli et al., 2012).The average length of CNC extracted from CPH cellulose is lower than the values reported for waste paper waste, *Mengkuang* leaves, mulberry bark, *Agave angustifolia* and Corn cob.

It is clear from this study that the use of stronger acid hydrolysis and ultrasonication in the isolation of CNC from cellulose produced CNCs with smaller dimensions than the earlier value reported for microcrystalline cellulose, MCC (with sizes within the range 286–1045 nm) extracted from CPH using only ultrasonication (Jimat and Jami, 2016). The CNC suspension was approximately 1.20 wt. % and the yield was ca. 25%. This yield is higher than 10.4% and 19% reported for CNC produced from *Mengkuang* fibre and waste paper respectively. In contrast, the % yield reported for corn cob (34.5%) and *Phormuim tenax* (35%) is higher than the valueobtained for CNCs in this study.

## 4 Conclusion

In this work cellulose nanocrystals were successfully extracted from CPH, an abundant lignocellulosic waste in Nigeria. This was achieved by acid hydrolysis after the CPH was subjected to an alkaline treatment and a bleaching chemical treatment. Amorphous materials, hemicelluloses and lignin were removed by the chemical treatments resulting in cellulose fibers with high cellulose 1 suitable for the production of cellulose nanocrystals. The extracted CNC showed increased crystallinity confirming the effectiveness of the alkaline and beaching treatments in the removal of hemicelluloses and lignin. SEM images showed significant changes in the fibre morphology with the fibers surface becoming smoother accompanied by reduction in size (from micrometer to nanometer) with progressive chemical treatments. CNC extracted from CPH displayed a rod-like morphology with diameter ranging from 10 to 60 nm and an average length of 95 nm. The results showed that CNCs can be successfully produced from CPH in a sustainable manner.

## Declarations

### Author contribution statement

Adebola Iyabode Akinjokun: Analyzed and interpreted the data; Wrote the paper.

Leslie Felicia Petrik: Contributed reagents, materials, analysis tools or data.

Aderemi Okunola Ogunfowokan: Conceived and designed the experiments.

John Ajao: Analyzed and interpreted the data.

Tunde Victor Ojumu: Conceived and designed the experiments; Contributed reagents, materials, analysis tools or data.

### Funding statement

This research did not receive any specific grant from funding agencies in the public, commercial, or not-for-profit sectors.

### Data availability statement

Data included in article/supplementary material/referenced in article.

### Declaration of interests statement

The authors declare no conflict of interest.

### Additional information

No additional information is available for this paper.
